# Macrophyta as a vector of contemporary and historical mercury from the marine environment to the trophic web

**DOI:** 10.1007/s11356-014-4003-4

**Published:** 2015-01-08

**Authors:** Magdalena Bełdowska, Agnieszka Jędruch, Joanna Słupkowska, Dominka Saniewska, Michał Saniewski

**Affiliations:** 1 Institute of Oceanography, University of Gdansk, Pilsudskiego 46, 81-378 Gdynia, Poland; 2 Institute of Meteorology and Water Management - National Research Institute, Maritime Branch, Waszyngtona 42, 81-342 Gdynia, Poland

**Keywords:** Macrophyta, Mercury, Bioaccumulation, Trophic chain, Coastal zone, Baltic Sea

## Abstract

Macrophyta are the initial link introducing toxic mercury to the trophic chain. Research was carried out at 24 stations located within the Polish coastal zone of the Southern Baltic, in the years 2006–2012. Fifteen taxa were collected, belonging to four phyla: green algae (*Chlorophyta*), brown algae (*Phaeophyta*), red algae (*Rhodophyta*) and flowering vascular plants (*Angiospermophyta*), and total mercury concentrations were ascertained. The urbanisation of the coastal zone has influenced the rise in Hg concentrations in macroalgae, and the inflow of contaminants from the river drainage area has contributed to an increase in metal concentration in vascular plants. At the outlets of rivers possessing the largest drainage areas in the Baltic (the Vistula and the Oder), no increases in mercury concentration were observed in macrophyta. Increase in environmental quality and a prolonged vegetative season results in the growing coverage of algae on the seabed and in consequence leads to rapid introduction of contemporary mercury and Hg deposited to sediments over the past decades into the trophic chain. Thriving phytobenthos was found to affect faster integration of Hg into the trophic web.

## Introduction

Mercury is considered to be one of the most dangerous contaminants of the environment. The adverse effect of Hg is related to its strong chemical and biological activity, as a result of which it is easily absorbed by organisms and spreads in the environment very rapidly. Mercury compounds become accumulated in tissues and can undergo biomagnification in organisms on higher trophic levels, reaching concentrations many times higher than in the environment itself (Förstner and Wittman 1981; Jackson 1998). Even small amounts of mercury in the system may lead to the disruption of biochemical processes, and it is also known to interfere with enzymatic and hormonal reactions, as well as protein and lipid biosynthesis. Hg is highly neurotoxic and can cause irreversible brain damage which may lead to autism in children and Alzheimer’s, Parkinson’s, schizophrenia and depression in adults (Zahir et al. 2005; Bose-O’Reilly et al. 2010).

People have used mercury for centuries, but emission of this metal into the environment increased significantly in the twentieth century as a result of industrial development. The main source of mercury pollution of the environment is fossil fuel combustion, but waste and sewage from various branches of industry also have a major impact. Owing to the highly adverse effects of Hg, numerous actions have been undertaken in recent decades aimed at replacing technologies involving mercury with more environmentally friendly alternatives. This has resulted in a much lower Hg inflow into the ecosystem.

Hg is introduced to the marine environment mainly via rivers, which transport pollutants from the drainage area and through atmospheric deposition. For many years, the Baltic Sea was considered to be one of the most polluted basins in the world (Wrembel 1997). However, according to a report by the Baltic Marine Environment Protection Commission, the mercury load introduced to the Baltic has decreased by 44 % since the 1990s (HELCOM 2010). In the case of the Baltic, the volume of Hg inflow is determined by the seven largest rivers that jointly introduce 70 % of the mercury (Wrembel 1993). Two of those large rivers—the Vistula and the Oder—flow through and end in Poland, their basins constituting 12 and 8 % of the Baltic’s drainage area, respectively (HELCOM 2004; Galassi et al. 2008). It is currently estimated that the volume of Hg inflow through rivers has fallen by about 16 % since 1990 (HELCOM 2010). In the same period of time, the level of atmospheric Hg deposition has, in turn, dropped by 24 % and the total volume of metals introduced into the Baltic in this way currently amounts to 3.1 t year^−1^ (Bartnicki et al. 2012). The reduction in the volume of Hg introduced into the Baltic Sea has led to decreased concentrations of this metal in these waters.

Organisms have long been used for the evaluation of environmental pollution of the sea. Plants and animals were first used as bioindicators in the 1960s, and benthic plants are considered to be one of the best pollution markers of aquatic environments (Stankovic et al. 2014). These plants absorb substances directly from their surroundings and, being attached to the ground, are permanently exposed to the environmental conditions (including water and sediment pollution) at their sites of growth. Another quality of phytobenthic organisms is their capacity to accumulate elements which do not perform any biological functions in their systems, of which mercury is one (Filipovic-Trajkovic et al. 2012). The accumulation and distribution of mercury in plants depends not only on the concentration of this metal in the environment and the physicochemical conditions but also on the species, the vegetative period, the size of the plant and the build of its root system and leaves (Nagajyoti et al. 2010). As a consequence, metal levels in various plant species occupying the same habitat may vary considerably (Wisłocka et al. 2006).

Macrophytobenthos also plays a key role in the biogeochemical circulation of chemical substances in the marine environment. Organic matter produced by benthic plants is consumed by numerous animals, thereby making these plants an important producer link in the trophic chain. Macrophyta are also one of the main Hg sources for their consumers: invertebrae and fish, both popular foods for humans (Stankovic et al. 2014). Mercury concentration levels in macrophyta and the ability of benthic plants to accumulate it are thus significant issues in studies on the transfer of this toxic metal from abiotic elements of the marine environment to higher levels of the trophic chain, including humans.

In the era of decreasing input of pollutants from anthropogenic sources, an attempt has been made to assess the role of macrophyta as a vector of Hg into marine trophic web, both the one entering the sea at present and that deposited to sediments over the past decades. Studies were conducted in the region of large and small river mouths, as well as away from river mouths (in the gulf waters and in the open sea).

## Materials and methods

### Study area

**Fig. 1:**
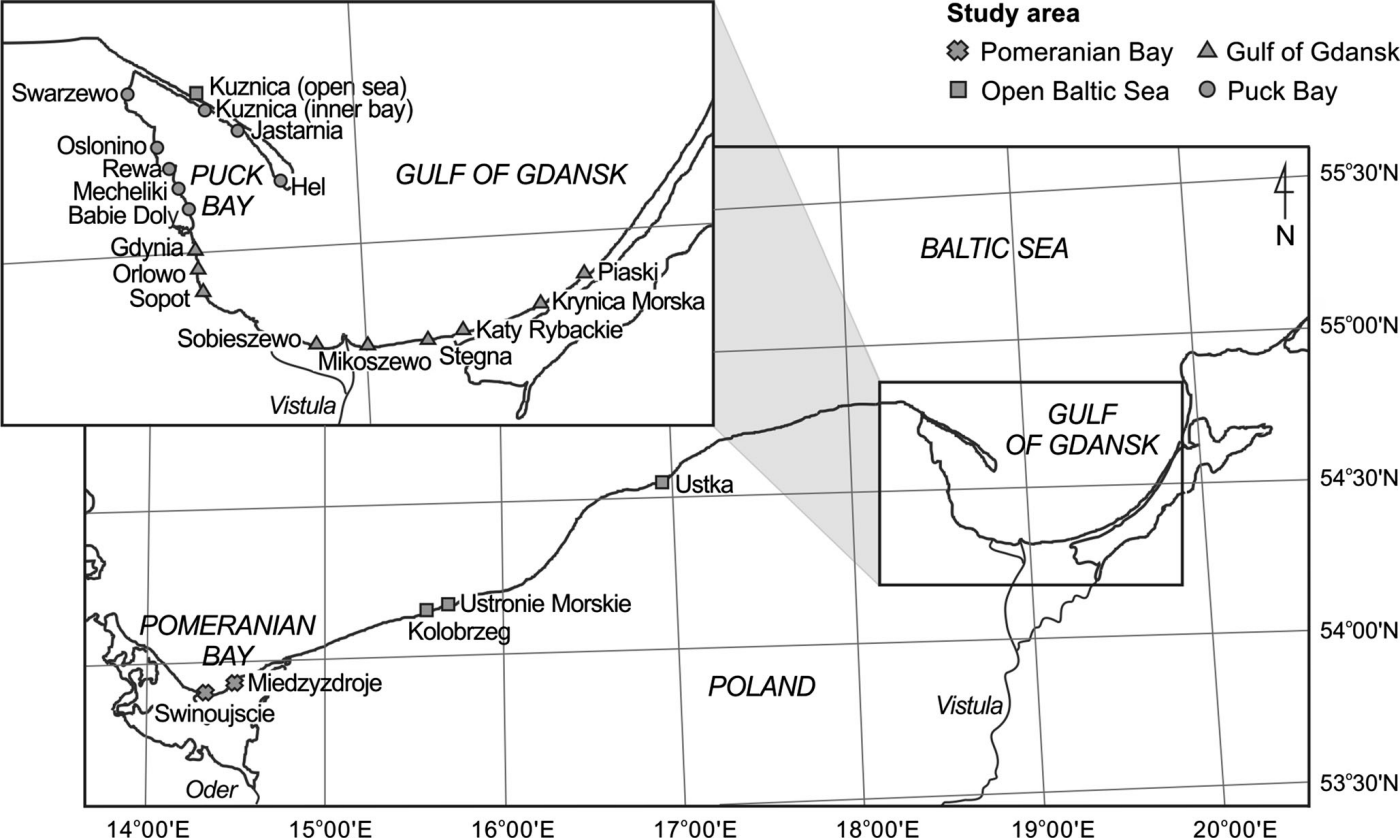
Location of sampling stations in the coastal zone of the Southern Baltic in 2006–2012

The study was conducted in the Southern Baltic, at 24 stations located in the Polish coastal zone (Fig. 1). The study area covered four sections of the Polish coastline: the Pomeranian Bay (Swinoujscie, 53° 54′ 28″ N, 14° 14′ 51″ E; Miedzyzdroje, 53° 55′ 44″ N, 14° 27′ 05″ E), an area of open sea near the coast (Kolobrzeg, 54° 10′ 40″ N, 15° 34′ 37″ E; Ustronie Morskie, 54° 12′ 55″ N, 15° 45′ 17″ E; Ustka, 54° 34′ 43″ N, 16° 52′ 09″ E; Kuznica (open sea), 54° 44′ 01″ N, 18° 34′ 56″ E), the Gulf of Gdansk (Gdynia, 54° 31′ 09″ N, 18° 32′ 22″ E; Orlowo, 54° 28′ 36″ N, 18° 32′ 56″ E; Sopot, 54° 26′ 31″ N, 18° 33′ 35″ E; Sobieszewo, 54° 20′ 11″ N, 18° 52′ 29″ E; Mikoszewo, 54° 20′ 02″ N, 18° 57′ 10″ E; Stegna, 54° 19′ 35″ N, 19° 06′ 44″ E; Katy Rybackie, 54° 20′ 22″ N, 19° 13′ 47″ E; Krynica Morska, 54° 22′ 57″ N, 19° 26′ 40″ E; Piaski, 54° 25′ 54″ N, 19° 36′ 05″ E) and Puck Bay (Hel, 54° 36′ 42″ N, 18° 48′ 29″ E; Jastarnia, 54° 41′ 58″ N, 18° 40′ 36″ E; Kuznica (inner bay), 54° 44′ 01″ N, 18° 34′ 56″ E; Swarzewo, 54° 45′ 31″ N, 18° 23′ 47″ E; Oslonino, 54° 40′ 08″ N, 18° 27′ 24″ E; Rewa, 54° 37′ 58″ N, 18° 30′ 37″ E; Mechelinki, 54° 36′ 32″ N, 18° 30′ 44″ E; Babie Doly, 54° 34′ 52″ N, 18° 32′ 23″ E).

The Pomeranian Bay is located in the southeastern part of the Baltic Sea, formed of a stretch of coast which is part Polish and part German. The environmental quality of this bay is strongly affected by the inflow of highly eutrophicated water from the Szczecin Lagoon, which nevertheless performs the function of a buffer, protecting the water of the Pomeranian Bay from pollutants flowing in from the drainage area of the River Oder. The condition of the Szczecin Lagoon and the coastal waters of the Pomeranian Bay, taking into account the evaluation of both biological and physicochemical parameters, is classified as bad (WIOŚ 2009). This is influenced by the contaminants accumulated in bottom sediments, as well as a constant inflow of pollutants from the drainage area and the vicinity of large urban centres, such as the Szczecin agglomeration (population 750,000). Moreover, Szczecin is a significant economical hub featuring a sea harbour, shipyards, numerous industrial facilities and wastewater treatment plants. An important influence on the water exchange between the Szczecin Lagoon and the Pomeranian Bay is exerted by the constant deepening of the water passage between Swinoujscie and Szczecin.

The Gulf of Gdansk is located in the northeastern part of the Baltic, between Poland and Russia. The main river entering this basin is the Vistula, the largest river flowing through Poland. The Gulf of Gdansk region abounds in anthropogenic pollution sources, and most of these are located within the Tri-city agglomeration, which is situated on the coast with a total population in excess of 1.2 million. The urban centres of this agglomeration are the harbour cities of Gdansk and Gdynia, which together feature numerous facilities related to shipbuilding, petrochemical and energy industry. The western part of the Gulf of Gdansk includes Puck Bay, a small area of water separated from the open sea by the Hel Peninsula. The Puck Bay ecosystem possesses the greatest biodiversity of any area within the Polish coastal zone of the Baltic. In 1978, the Inner Puck Bay became the first sea area to fall under protection as part of the Coastal Landscape Park and, in 1992, it was included in the Baltic Sea Protected Areas (BSPA). It also features on the World Wide Fund for Nature list (WWF).

The stations situated in the open sea area (Kolobrzeg, Ustronie Morskie, Ustka, Kuznica) were located far away from large industrial facilities, in proximity only to small holiday destinations whose populations amount to between 630 (Kuznica) and 47,000 inhabitants (Kolobrzeg).

### Sample collection

Benthic plant samples were collected in the coastal zone in the years 2006–2012. Macrophytobenthos was collected by hand at depths of about 1 m into polyethylene ziploc bags, making sure that each sample contained an entire plant. The collected material was taken to the laboratory, cleansed of epiphytes, organic and mineral particles, and then rinsed in distilled water. Taxonometric analysis was also carried out. The representatives of two species were additionally separated into particular components—roots, stems and leaves in the case of *Potamogeton pectinatus*, and rhizoids and stipes in the case of *Furcellaria lumbricalis*. The plant material was stored at −20 °C until analysis and, immediately prior to testing, was lyophilised and homogenised.

Total mercury concentration (Hg_TOT_) in macrophytobenthos samples was measured using atomic absorption spectrometry (AAS) on an AMA-245 advanced mercury analyser (Altec Ltd., Czech Republic), following thermal decomposition in pure oxygen. A quality check on the method involved analysing the collected samples in three repeats, together with analysis of certified reference material: BCR-27 (*Ulva lactuca*) and BCR-60 (aquatic plant). The method was characterised by high recovery, amounting to 96–98 %, the relative standard deviation was no higher than 5 %, and the limit of quantification was 0.01 ng g^−1^ dw.

On the basis of the obtained results for total mercury concentrations in macrophytobenthos and its surroundings, it was possible to calculate the bioconcentration factor, which is a parameter reflecting the ability of plant organisms to absorb mercury from water. The bioconcentration factor (BCF) was calculated using the following formula (Szefer and Żbikowski 2010):

$$ \mathrm{B}\mathrm{C}\mathrm{F}=\frac{{\mathrm{C}}_{\mathrm{X}}}{{\mathrm{C}}_{\mathrm{W}}} $$

where

C_x_: Mercury concentration in wet mass of the organism (ng kg^−1^ ww)
C_w_: Mercury concentration in the surrounding water (ng dm^−3^)

In order to determine the mercury BCF for stems, leaves and stems of benthic organisms, near-bottom water was taken into account as the surrounding environment, while in the case of roots and rhizoids, pore water was examined.

Statistical analysis and graphic representation of results was carried out using Statistica 10 software (StatSoft), and the analysed data was found to be non-parametric (Shapiro-Wilk test, *p* < 0.05). In order to determine the relevance of differences, the Mann-Whitney *U* and Kruskal-Wallis tests were used at relevance level of *p* = 0.05.

## Results and discussion

The plant material collected in the Polish coastal zone of the Baltic Sea in the years 2006–2012 consisted of both macroalgae and vascular plants. Among the collected samples, 15 taxa belonging to four phyla were identified—green algae: *Chara* sp., *Cladophora* sp., *Ectocarpus siliculosus*, *Elodea canadensis* and *Enteromorpha* sp.; brown algae: *Chorda filum*, *Fucus vesiculosus*, *Pylaiella littoralis* and *Sphacelaria cirrosa*; red algae: *Furcellaria lumbricalis* and *Polysiphonia* sp.; and flowering vascular plants: *Potamogeton pectinatus*, *Ruppia maritima*, *Zannichellia palustris* and *Zostera marina*. The macrophytobenthic species identified were those typically found in the Southern Baltic coastal region (Pliński and Florczyk 1993; Schubert and Krause 2002).

**Fig. 2:**
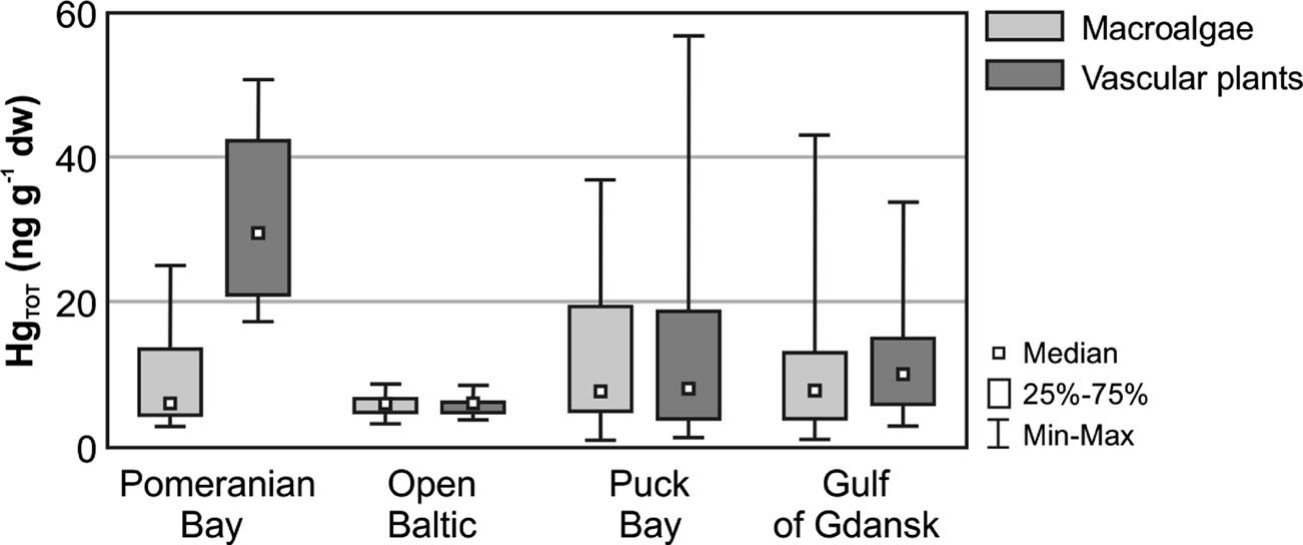
Concentration range of total mercury (Hg_TOT_) in macrophytobentos: macroalgae and vascular plants, in different regions of the coastal zone of the Southern Baltic in 2006–2012

In all the analysed macrophytobenthos samples, total mercury concentrations (Hg_TOT_) were above the limit of quantification (Fig. 2). The obtained values ranged between 0.8 and 57.2 ng g^−1^ dw (median 7.6 ng g^−1^ dw, mean 10.9 ng g^−1^ dw, *n* = 248), while mercury concentrations in vascular plants (median 8.5 ng g^−1^ dw, mean 12.2 ng g^−1^ dw, *n* = 83) and macroalgae (median 7.3 ng g^−1^ dw, mean 10.2 ng g^−1^ dw, *n* = 165) did not demonstrate differences that were statistically relevant (Mann-Whitney *U* test, *p* = 0.21). In vascular plants, Hg_TOT_ concentrations ranged between 1.3 ng g^−1^ dw for sago pondweed *Potamogeton pectinatus* and 57.2 ng g^−1^ dw for eelgrass *Zostera marina*. In macroalgae, the obtained values ranged from 0.8 ng g^−1^ dw, determined in gut weed *Enteromorpha* sp., to 43.3 ng g^−1^ dw, as determined for black carrageen *Furcellaria lumbricalis* (Table 1, Fig. 2).

**Table 1:** Median values and concentration ranges of total mercury (Hg_TOT_, ng g^−1^ dw) in macroalgae (*Chlorophyta*, *Rhodophyta* and *Phaeophyta*) and vascular plants (*Angiospermophyta*) from the Polish coastal zone of the Southern Baltic in 2006–2012

Region	*Chlorophyta*	*Phaeophyta*	*Rhodophyta*	*Angiospermophyta*
Pomeranian Bay	4.7 (3.0–14.3)	6.0 (4.6–7.3)	25.4	29.6 (17.7–50.8)
Open Baltic sea	5.1 (3.8–7.9)	6.3 (4.4–8.4)	7.6 (6.0–8.7)	6.0 (3.7–8.5)
Puck Bay	10.1 (0.8–37.1)	5.2 (4.3–6.8)	11.0	8.3 (1.3–57.2)
Gulf of Gdansk	7.1 (0.9–29.9)	9.1 (4.9–15.6)	7.8 (1.3–43.3)	9.3 (2.8–33.8)

Hg_TOT_ concentrations in the macrophytobenthos of the Southern Baltic coastal zone were found to be much lower in 2006–2012 than those determined in 1995–1998 in the Puck Bay region (Boszke et al. 2003). This is mainly due to the fact that mercury inflow into the Baltic has decreased as the result of a systematic reduction of metal emissions into the environment (HELCOM 2004, 2010). This resulted in a drop in Hg concentration in sea water (Wrembel 1997; Saniewska et al. 2010) and, as a consequence, also in macroalgae. The Hg concentrations in macroalgae presented in this paper were from three to ten times lower than those determined in previous years (Boszke et al. 2003). In the case of vascular plants, which are rooted to the seabed, the decrease in metal concentration was lower with Hg_TOT_ values coming in at 2–2.5 times lower than those from the 1990s. This may be attributed to the fact that mercury concentration in surface sediments is not reduced as rapidly as in surface water (Jędruch et al. 2013b).

### Geographic variation of Hg_TOT_ concentration

Total mercury concentrations determined in macrophytobenthos collected in the Polish coastal zone were characterised by variability in each study area. Hg_TOT_ concentrations in the organisms from the open sea coastal area were lower in terms of statistical relevance than in the organisms collected in the Pomeranian Bay, the Gulf of Gdansk or Puck Bay (Kruskal-Wallis test, *p* = 0.03), and also had the most limited range (Fig. 2). In vascular plants, Hg_TOT_ concentrations in the coastal zone of the open sea (median 6.0 ng g^−1^ dw) were lower by 35 % when compared to the other, more sheltered parts of the basin (median 9.2 ng g^−1^ dw). In macroalgae (median 6.1 ng g^−1^ dw), the obtained values were 20 % lower than those from within the bays (median 7.6 ng g^−1^ dw). These differences were mainly due to mercury concentration levels in the surrounding environment (Broekart et al. 1990) and while the concentration of this metal determined in the coastal water of the open sea was similar to values observed in the Baltic Proper (Pempkowiak et al. 1998; Murawiec et al. 2007; Bełdowski et al. 2009), Hg_TOT_ concentrations found within the Gulf of Gdansk and Puck Bay were nearly twice as high (Saniewska et al. 2010). A similar tendency was also observed for the concentration of heavy metals in zooplankton of the Southern Baltic (Pempkowiak et al. 2006). The lower mercury concentrations noted in the coastal waters of the open sea were influenced by, among other factors, the distance from anthropogenic sources of metal emission and outlets of large rivers—total annual run-off from rivers flowing directly into the Baltic Proper is seven times smaller than run-off from the River Vistula and four times smaller than that of the Oder (Bogdanowicz 2009; Niemirycz 2011).

In the Pomeranian Bay, the differences between total mercury concentrations in vascular plants (median 29.6 ng g^−1^ dw) and macroalgae (median 6.0 ng g^−1^ dw) were statistically relevant (Mann-Whitney *U* test, *p* = 0.03), while concentrations in the higher plants exceeded values found in the other studied regions of the Southern Baltic by over threefold (Fig. 2). Higher Hg_TOT_ concentrations in vascular plants, which absorb chemical substances additionally via the root system (Lack and Evans 2005), may be attributed to increased metal concentration in surface sediment compared to other areas of the Polish coastline. Mercury is introduced to the Pomeranian Bay primarily by the River Oder and the waters of the Szczecin Lagoon, one of the most polluted regions of the Polish coast in terms of heavy metals (Glasby et al. 2004; Szefer et al. 2009). Concentrations of these metals in macrophytes have a significant influence on the levels found in organisms occupying higher trophic levels: fish and marine birds. Owing to their placement in the marine food web and the fact that mercury is introduced into their systems mainly through the alimentary canal, birds are held to be good indicators of environmental mercury pollution (Kalisińska et al. 2004). Research carried out in 2004–2005 in the Szczecin Lagoon confirmed the existence of a positive correlation between mercury concentrations in the ingesta and in the tissues of the mallard duck *Anas platyrhynchos*, whose diet is based on phytobenthos (Lisowski 2009). Furthermore, Hg_TOT_ concentrations in the soft tissues of *A. platyrhynchos* (liver 270 ng g^−1^ dw, kidney 250 ng g^−1^ dw, muscle 130 ng g^−1^ dw) were nearly twice as high as those determined for the black-headed gull *Larus ridibundus* from the Gulf of Gdansk, whose diet is composed largely of fish, in 2009–2012 (liver 130 ng g^−1^ dw, kidney 162 ng g^−1^ dw, muscle 60 ng g^−1^ dw) (Kalisińska et al. 2013; Szumiło et al. 2013). High mercury concentrations in *A. platyrhynchos* are indicative of a level of mercury pollution in the Pomeranian Bay which could lead to deterioration of bird’s health: disruptions in visual-motor coordination, spatial orientation or a significant reduction in terms of breeding success (Scheuhammer et al. 2008). This is proven by concentrations in the soft tissues of the mallard duck *A. platyrhynchos* exceeding the threshold value of 250 ng g^−1^ dw, above which adverse effects of mercury can be observed in organisms (Kalisińska et al. 2013). This is particularly important in the case of *A. platyrhynchos* as it is the most common gamebird consumed in Poland, the number of specimens shot annually reaching up to 120,000 (Książkiewicz 2006).

Increased mercury concentrations, in comparison with the open sea coastal area, were also observed in vascular plants (median 10.5 ng g^−1^ dw) in the Gulf of Gdansk region (Fig. 2). This was mainly as a result of close proximity to the outlet of the Vistula, the largest Polish river and the second largest river flowing into the Baltic (after the Neva) in terms of drainage area size and level of run-off (HELCOM 2004). As noted in previous studies, mercury concentrations in the Vistula (median 6.3 ng dm^−3^, mean 7.3 ng dm^−3^) are slightly higher than the global average for unpolluted rivers (mean 5.0 ng dm^−3^) (Mason et al. 1994; Saniewska et al. 2014). Moreover, owing to its large flow of water, the Vistula contributes a significant mercury load to the Gulf of Gdansk, including the bioavailable dissolved form of the metal which is washed off the drainage area (Saniewska et al. 2014). Additional sources of Hg are storm drain outlets, which discharge the run-off from highly urbanised areas such as the Tri-city agglomeration (Saniewska 2013). Hg_TOT_ concentrations determined in macroalgae (median 7.6 ng g^−1^ dw), absorbing mercury mainly from the near-bottom water, were, as in the Pomeranian Bay, lower than in the more advanced plants. This is probably related to the fact that mercury concentrations determined in pore water were almost twice as high as in surface water (Bełdowska et al. 2013b).

The total mercury concentration in the rooted vascular plants from Puck Bay (median 8.3 ng g^−1^ dw) was 20 % lower than in the Gulf of Gdansk (Fig. 2). This is likely to be connected to the physicochemical qualities of Puck Bay, which are conditioned mainly by shallow water depth and the accumulation of organic matter. As a consequence, the near-bottom zone of the bay is characterised by low oxygen levels and periodical occurrence of hydrogen sulfide. In reductive conditions such as these, precipitation of the insoluble mercury sulfide may have occurred in sediments, resulting in a reduction of mercury bioavailability for phytobenthos. Numerous studies confirm that the concentration of mercury dissolved in pore water increases with a rise in Eh potential and decreases as H_2_S concentration grows. Moreover, it has been found that the insoluble HgS is one of the main metal forms in the sediment of Puck Bay (Bełdowski and Pempkowiak 2007; Bełdowska et al. 2013c; Jędruch et al. 2013b). In the case of Hg_TOT_ concentration in the macroalgae of Puck Bay (median 7.9 ng g^−1^ dw), no statistically relevant differences were observed in relation to the values determined in the Gulf of Gdansk (Mann-Whitney *U* test, *p* = 0.16) (Fig. 2). Although Puck Bay experiences little anthropogenic influence and is at a considerable distance from the outlet of the River Vistula, the rivers flowing into it nevertheless turned out to be a significant source of the bioavailable mercury form. These watercourses, transporting mercury washed off urban areas, cultivated fields and forests could have had a significant influence on metal concentrations close to their outlets (Saniewska 2013).

### Mercury in *Zostera marina*

**Fig. 3:**
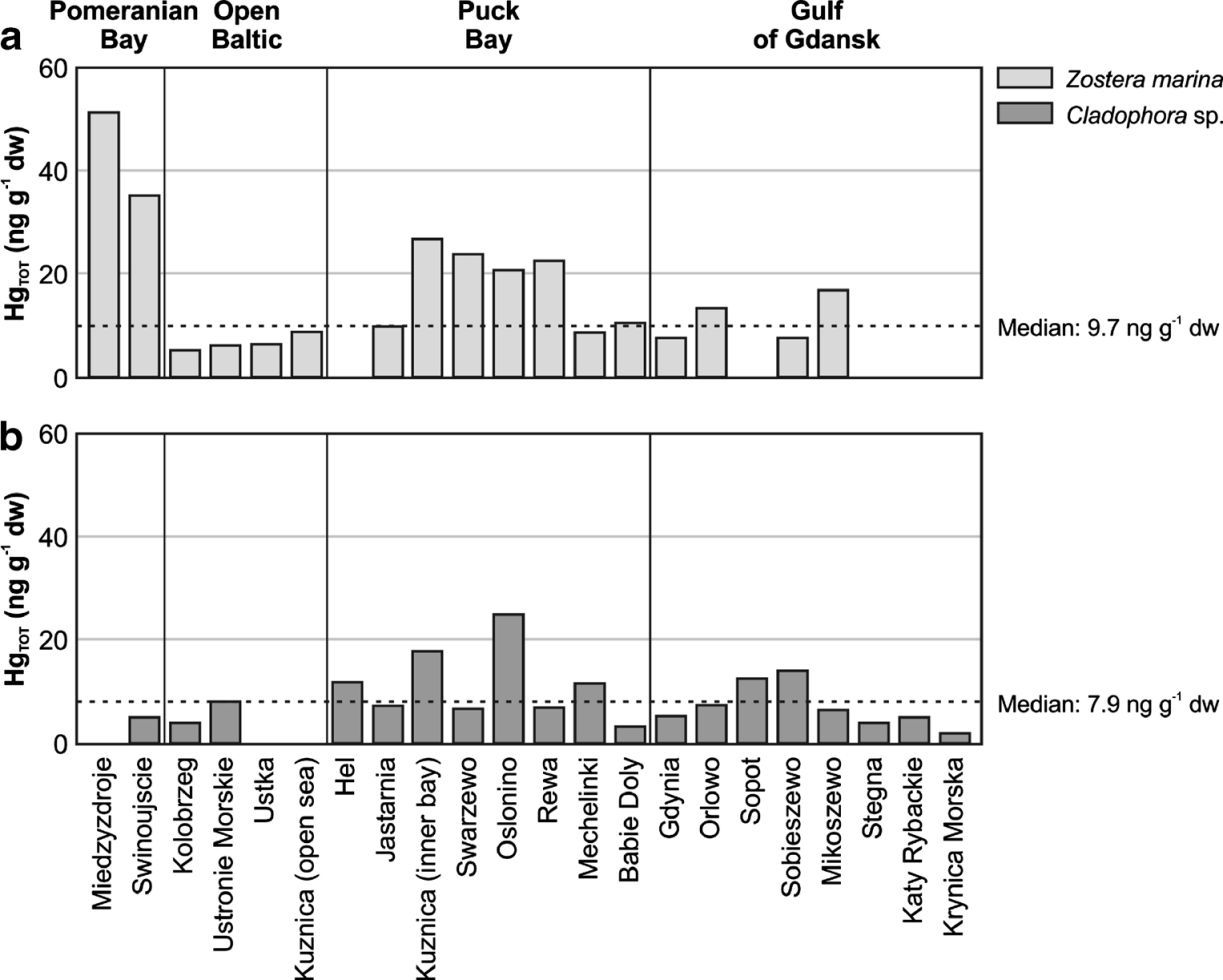
Median values of total mercury (Hg_TOT_) concentration in *Zostera marina* (**a**) and *Cladophora* sp. (**b**) at different sampling stations in the coastal zone of the Southern Baltic in 2006–2012

The influence of increasing distance from river outlets on Hg concentration levels in macrophytobenthos was particularly noticeable in one vascular plant, *Zostera marina*, collected at most of the test stations. Hg_TOT_ concentrations in this seagrass showed statistically relevant differences at the particular test stations (Kruskal-Wallis test, *p* = 0.02), and the highest values were determined close to river outlets (Fig. 3a). Studies on mercury speciation in suspended matter, carried out by Bełdowski (2004) in the studied region, showed that the highest proportion of labile Hg forms in suspended matter occurred in the vicinity of river outlets. The maximum Hg_TOT_ concentrations were found in the *Zostera marina* samples collected in Miedzyzdroje (median 50.8 ng g^−1^ dw) and Swinoujscie (median 34.9 ng g^−1^ dw) (Fig. 3a), both located close to the mouth of the River Oder, the second largest river in Poland and the third largest river flowing into the Baltic in terms of drainage area (HELCOM 2004). The fact that the water from the Oder flows into the Szczecin Lagoon first causes a large proportion of suspended matter to deposit in the coastal zone, resulting in a rise in pollution. Mercury concentrations which were higher than the Hg median for the study area were also found in the seagrass collected at the Mikoszewo station, located a short distance from the River Vistula outlet. Despite it being the largest river in Poland, however, Hg concentrations in the area of its outlet were not the highest. This was related, among other things, to its strong current transporting the metal into further regions of the sea (Bełdowski and Pempkowiak 2007; Damrat et al. 2013). Even though the mercury load introduced to the Baltic by large rivers is considerable, small rivers can also constitute an ecological problem on a local scale, particularly in the coastal zone (Cyberski et al. 1993; Niemirycz 2011). This is indicated by increased concentrations of the metal in *Zostera marina* in the outlet areas of smaller rivers, at the stations in Swarzewo (River Plutnica), Oslonino (River Gizdepka), Rewa (River Reda, Zagorska Struga, drainage canals) and Orlowo (River Kacza) (Fig. 3a). Most mercury of river origin is related to suspended matter and, in the case of small rivers, this tends to undergo sedimentation in the coastal zone close to the outlet (Cossa and Martin 2001; Saniewska 2013; Saniewska et al. 2014). Mercury from coastal surface sediments may then be released into pore water as a result of its oxidation to soluble sulfates (Bełdowski et al. 2009), enabling it to be absorbed by rooted vascular plants.

### Mercury in *Cladophora* sp.

Proximity to point sources of dissolved mercury had an effect on increased Hg_TOT_ concentrations in macroalgae, exemplified here by *Cladophora* sp., which was collected at most test stations. Concentrations of Hg in this alga displayed statistically relevant spatial variability (Kruskal-Wallis test, *p* = 0.04), and the highest concentrations were found in the Oslonino area (median 24.7 ng g^−1^ dw) (Fig. 3b). This probably resulted from the presence in the water of mercury in its bioavailable form and its subsequent bioaccumulation in the macroalgae. The mercury concentrations determined in surface water, plankton organisms, surface sediments and pore water in the Oslonino coastal zone were also found to be higher in comparison with the rest of the Gulf of Gdansk coast (Bełdowska et al. 2013a, d). This may be attributed to the inflow of Hg with the River Gizdepka (Saniewska 2013), and the sediments derived from eroded cliff into the sea. As shown by studies carried out in 2011–2013, a significant load of mercury deposited on land over the course of many years is introduced into the Gulf of Gdansk as a result of coastal erosion (Jędruch et al. 2013c). The level of Hg concentration in the coastal area of Oslonino was also influenced by the particular physicochemical conditions of the test station, which is located in a small bay where intense accumulation of decomposing organic matter takes place as a result of shallow depth and low water dynamics.

Increased Hg_TOT_ concentrations were also found in *Cladophora* sp. samples collected in the Hel region (Fig. 3b), and this may have resulted from the proximity of the station to fishing and navy ports. Mercury concentrations in excess of the median value were also detected in Sopot, close to storm drain outlets which introduce pollutants from the Tri-city agglomeration into the basin (Cyberski et al. 1993; Saniewska 2013), and in Mechelinki, where the source of dissolved mercury was probably wastewater discharged from the collector of the Debogorze treatment plant. The influence of this plant on the inflow of bioavailable mercury into the basin has already been confirmed by analyses of metal speciation in surface sediments in the Mechelinki region, which indicate a large proportion of dissolved forms of mercury, including those related to organic matter—fulvic and humic acids (Bełdowski and Pempkowiak 2007).

High concentrations of this metal were also determined in the area of Sobieszewo and were probably related to two bird sanctuaries (Ptasi Raj and Mewia Lacha) located on the island, hosting many different bird species. Mercury can be removed from the systems of birds nesting in these sanctuaries through moulting, excretion with guano or deposition in eggs. These congregations of birds and the consequent presence of guano have become sources introducing toxic substances into the sea water, as confirmed by Falkowska et al. (2007). Additionally, research has shown that a female bird can remove about 5 % of the mercury consumed with food in the laying of eggs (Falkowska et al. 2013a), thus re-introducing Hg back into the trophic chain.

### Mercury in the particular parts of macrophytae

**Fig. 4:**
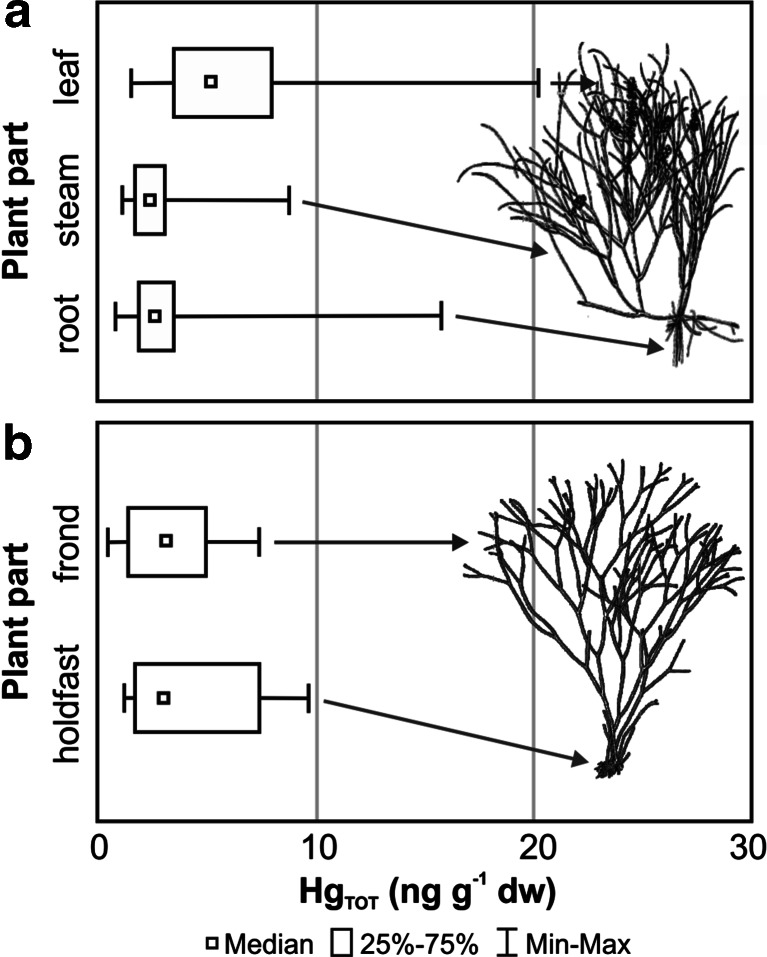
Concentration range of total mercury (Hg_TOT_) in the different parts of *Potamogeton pectinatus* (**a**) and *Furcellaria lumbricalis* (**b**) from the coastal zone of the Southern Baltic in 2006–2012

Trace metals can be absorbed by benthic plants both through the roots, from pore water, and through leaves and stems, from the surrounding water (Conquery and Welbourn 1994). The diverse ability to absorb mercury from the environment is exemplified here by the vascular plant *Potamogeton pectinatus*, from the Oslonino region, and the red alga *Furcellaria lumbricalis*, collected in the Orlowo region. Both of these species of macrophytobenthos were characterised by statistically relevant variability of mercury BCF (U Mann–Whitney test, *p* = 0.01). In *Potamogeton pectinatus*, the BCF from the near-bottom water (median 345.9) was much higher than in *Furcellaria lumbricalis* (median 204.4), indicating a greater ability to accumulate Hg in vascular plants compared to macroalgae. What is more, in vascular plants mercury absorption from the near-bottom water through the parts of the plant that grow above the sediment, occurred seven times more effectively than metal absorption from the pore water through the roots (median 45.2) (Mann-Whitney *U* test, *p* = 0.01). This is also confirmed by the Hg_TOT_ levels determined in the particular parts of *Potamogeton pectinatus*, concentrations in the leaves (median 5.1 ng g^−1^ dw, *n* = 43) being significantly higher than those found in the roots (median 2.6 ng g^−1^ dw, *n* = 29) and stems (median 2.4 ng g^−1^ dw, *n* = 30) (Kruskal-Wallis test, *p* = 0.00) (Fig. 4a). Both the highest mercury concentrations and the highest BCF value (median 395.2) were determined in the leaves of *Potamogeton pectinatus* and resulted from simultaneous absorption of metal from the near-bottom water and transportation into the leaves of Hg absorbed through the roots from pore water (Jackson 1998; Lack and Evans 2005). Increasing concentrations of this metal in the leaves of marine vascular plants, in comparison with the rest of the plant, have also been observed by other researchers (Pergent-Martini 1998; Göthberg et al. 2004; Lafabrie et al. 2011). Higher mercury bioconcentration was noted in the aboveground parts of *Potamogeton pectinatus*, and this was mainly due to the greater absorption surface of the leaves. However, it could also have resulted from the defensive action of the organism, which involves restricting the penetration of metal ions into the roots if mercury concentration in sediments increases (Jędruch et al. 2013b). In the case of macroalgae, which are not rooted to the ground but only attached to it with the use of rhizoids, mercury is absorbed mainly from the surrounding water. This explains the lower BCF values calculated for *Furcellaria lumbricalis* in comparison with a vascular plant. Furthermore, no statistically relevant differences were found for the BCF values of the rhizoids (median 192.3) and the stipe of the red alga (median 246.4) (Mann-Whitney *U* test, *p* = 0.70). Hg_TOT_ concentration was also evenly distributed in the constituent parts of the plant (Mann-Whitney *U* test, *p* = 0.31), with the level measured in the rhizoids (median 2.9 ng g^−1^ dw; *n* = 11) being similar to that of the stipe (median 3.0 ng g^−1^ dw, *n* = 23) (Fig. 4b). This may be explained by the fact that thallus tissues in macroalgae are not very diversely built, resulting in uniform absorption of mercury through the rhizoids and stipe (Lack and Evans 2005). The differing ability of vascular plants and macroalgae to absorb mercury from the surrounding environment may have an effect on the circulation of this metal in the marine environment. Higher Hg concentrations in vascular plants suggest that, in areas where the seabed is covered in those organisms, mercury will accumulate more effectively than in other areas.

### Mercury in macrophyta biomass from Puck Bay

The role of macrophytobenthos in the process of introducing pollutants into the trophic chain depends on the state of the marine environment, which can have an influence on conditions limiting the development of seabed flora. In the years 1950–1980, as a result of factors including eutrophication of the basin, there was a deterioration in water quality which caused the macrophytobenthos coverage of the Baltic seabed to be considerably reduced. At the same time, areas covered in benthic plants decreased by as much as 90 % in some parts of the basin (Boström et al. 2014). In recent years, however, some restoration of the seagrass bed of the Baltic has been observed, including within the Polish coastal area (Kruk-Dowgiallo 1991; Gic-Grusza et al. 2009). This has been confirmed by, among others, the results of an ecological assessment study into the condition of seabed flora, conducted by Saniewski (2013) in 2011, in which the rating ranged from moderate inside the bays to good in the shallow area of the central coast. Those results also indicated that some species previously considered as rare (i.e., the brown alga *Polysiphonia* sp. and the red alga *Furcellaria lumbricalis*) had previously covered quite a large part of the seabed in the Polish coastal zone of the Baltic and that their proportion in the total macroalgae biomass had in some areas exceeded 80 % (Osowiecki et al. 2009; Saniewski 2013). This is of particular importance as, in the present study, brown and red algae were characterised by significantly higher Hg_TOT_ concentrations than macroalgae from the Chlorophyta group (Kruskal-Wallis test, *p* = 0.00) (Table 1). Increased Hg_TOT_ concentrations in brown and red algae can be explained by the presence of charged polysaccharides in the cell walls (Mamboya 2007). As shown by research, the occurrence of alginates and sulphades fucans with higher affinity for cations, and the presence of physodes containing phenolic compounds, can influence the ability of macroalgae to absorb metals from the surrounding environment (Andrade et al. 2002; Farina et al. 2003; Salgado et al. 2005). Research carried out in the coastal zone of the Baltic also indicated a greater ability of red and brown algae, in comparison with green algae, to bioaccumulate radionuclides, as proven by measurement of the ^137^Cs and ^90^Sr isotopes in their tissues (Zalewska and Saniewski 2011; Saniewski 2013). This shows how important these groups are to the circulation of pollutants in the marine environment.

**Fig. 5:**
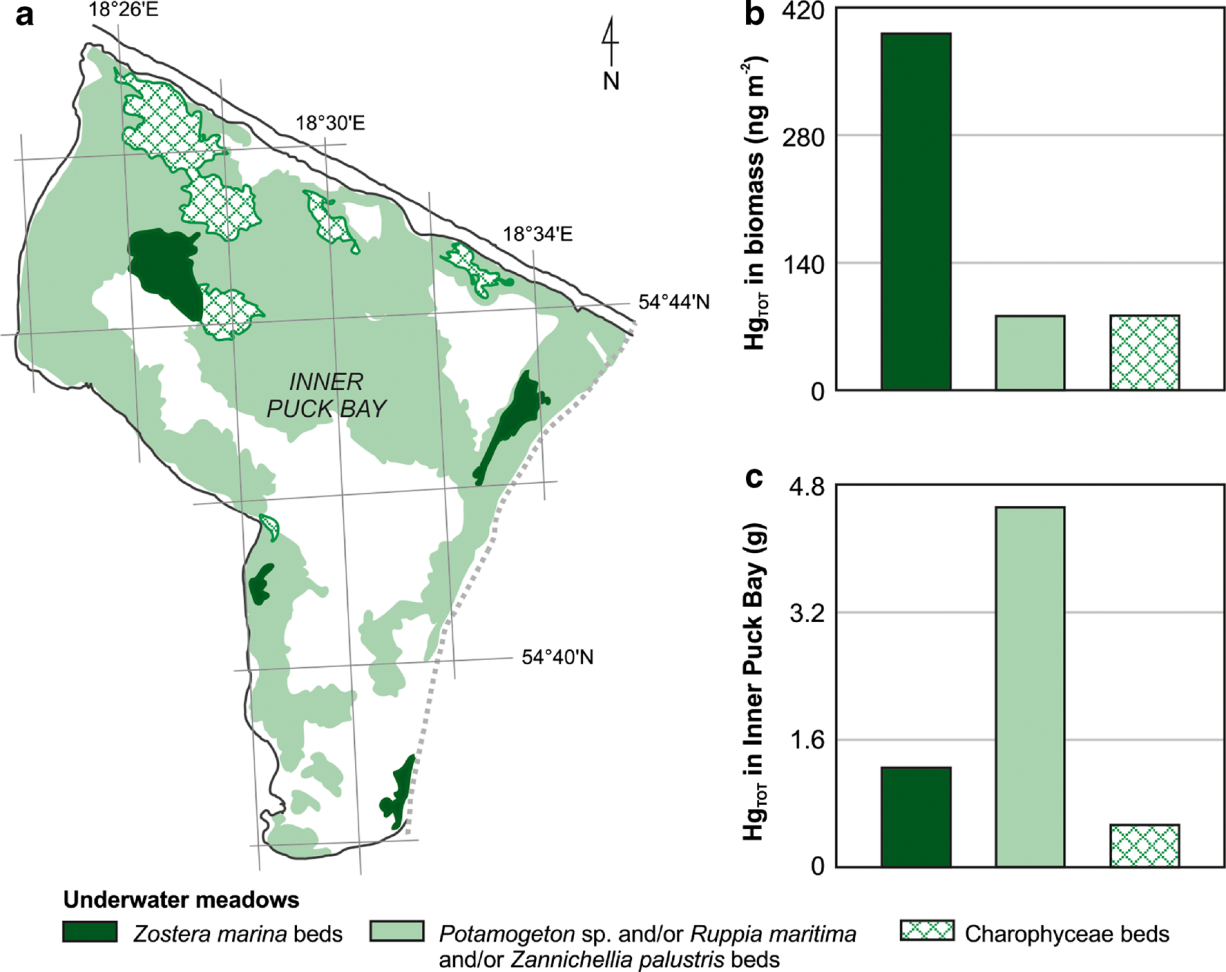
Distribution of macrophyte beds in the Inner Puck Bay, based on Gic-Grusza et al. (2009) (**a**), and median values of total mercury (Hg_TOT_) in biomass in different types of meadows (**b**) with the amount of Hg_TOT_ accumulated by them (**c**)

A good quality of water is also reflected by the presence of vascular plants, which are sensitive to eutrophication (Krause-Jensen et al. 2008), and in many areas of the Baltic, the seagrass bed is much more widely distributed than previously reported (Boström et al. 2003, 2014). In the Polish coastal zone, clusters of vascular plants cover the seabed especially in the Inner Puck Bay (Fig. 5) and the most valuable species present, in ecological terms, is *Zostera marina*, the clusters of which, according to research carried out in 2007–2009, covered 3 % of the basin’s area (Węsławski et al. 2013) (Fig. 5). The area of the Puck Bay seabed covered in *Zostera marina* has been increasing in recent years and is now six times larger than it was in the 1980s (Kruk-Dowgiallo 1991; Gic-Grusza et al. 2009; Węsławski et al. 2013). Research carried out in 2010–2011 found the proportion of this species present in the total plant biomass of the seagrass bed (approximately 50 g dw m^−2^) to be as high as 80 %, while the proportions of particular parts in the biomass of the species were found to be about 60 % for aboveground parts and 40 % for belowground (Jankowska et al. 2014). Taking into account the Hg concentration median for this species, it was estimated that in 1 m^2^ of the seabed covered in seagrass, up to 390 ng of total mercury was accumulated, out of which 215 ng m^−2^ was accounted for by the above-sediment parts of the plant (median Hg_TOT_ concentration 8.9 ng g^−1^ dw), and 175 ng m^−2^ by the roots (median Hg_TOT_ concentration 10.9 ng g^−1^ dw) (Fig. 5). A sizeable portion of the sea bottom in Puck Bay (53 %) is covered in an assortment of brackish-water flora: *Potamogeton pectinatus*, *Ruppia maritima* and *Zannichellia palustris* (Węsławski et al. 2013). Their combined biomass was twice as high as in the case of seagrass and amounted to about 16 g dw m^−2^ (Brzeska and Saniewski 2012) which, taking into account mercury concentration in plant tissues (median Hg_TOT_ concentration 5.0 ng g^−1^ dw) translates to 81 ng of metal per 1 m^2^ of the seabed (Fig. 5). Another valuable feature of the Inner Puck Bay are clusters of *Charophyta*, covering 6 % of the sea bottom area of this basin (Węsławski et al. 2013). Despite their low biomass, amounting to about 6 g dw m^−2^ (Brzeska and Saniewski 2012), in 1 m^2^ of seabed covered in charophytae, the level of mercury accumulation (82 ng m^−2^) is similar to that observed in clusters of assorted vascular plants (Fig. 5). This is related to the presence of higher mercury concentrations in those organisms (median Hg_TOT_ concentration 13.6 ng g^−1^ dw). The mercury accumulated in marine plants is accessible to organisms from a higher level of the trophic chain. Hg_TOT_ values in macrophytobenthos biomass, ranging from double figures to several hundred nanograms per 1 m^2^ of the sea bottom, were an order of magnitude lower than the total mercury concentrations determined in zoobenthos biomass in Puck Bay (Jędruch et al. 2013a). Moreover, Hg_TOT_ values in zoobenthic biomass were found to be the highest in samples containing the largest proportion of *Idotea* sp.—the main consumer of seagrass in the Baltic (Baden et al. 2010).

Taking into consideration the concentration of mercury in macrophytobenthic biomass and the area of sea bottom covered by it (Gic-Grusza et al. 2009; Węsławski et al. 2013), it was possible to calculate the metal load accumulated in these plants (Fig. 5). In the Inner Puck Bay, seabed flora may jointly contain over 6 g of mercury, of which widespread assorted clusters of *Potamogeton pectinatus*, *Ruppia maritima* and *Zannichellia palustris* account for 4.5 g, clusters of *Zostera marina* seagrass account for 1.3 g, and Charophyceae account for 0.5 g (Fig. 5). Due to a complete lack of exact data concerning macrophytobenthic biomass in the basin, the calculated load does not take into account other plant clusters, including macroalgae covering the hard seabed, numerous string algae or other vascular plant species. The increased occurrence and subsequent rise in biomass of vascular plants which has been observed in Polish coastal waters in recent years may therefore play a significant role in delivering heightened levels of mercury to the trophic web and increasing concentrations of this metal in more advanced organisms. Another factor influencing this phenomenon is the prolonged vegetative season, which is related to climate changes in the Polish coastal area (Kożuchowski 2009; Bełdowska et al. 2013b). The absence of ice cover on the bay allows Hg absorption from water and sediments to continue for longer, in turn causing the metal to remain in the trophic chain for an extended period of time.

### Mercury in beach-cast macophytobenthos biomass

In many areas of the Baltic, owing to the distribution of currents and the shape of the coastline, large amounts of macrophytobenthos accumulate in the coastal zone or are cast onto beaches. During the summer season in the Gulf of Gdansk, macroalgae accumulation along 1 km of beach can range from several dozen up to as much as 800 t (Filipkowska et al. 2008; Blidberg and Gröndahl 2012). Taking into account the median for Hg_TOT_ concentration 7.6 ng g^−1^ dw, it was estimated that 1 km of beach can accumulate over 6 g of mercury over the course of a few months. Studies into the speed of coast alternations in the Southern Baltic region show that 39 % of the length of the Polish coast is of an accumulative nature (Dubrawski and Zawadzka 2006). This equates to about 200 km of coastline along which processes favourable to the accumulation of phytobenthos may occur. In the summer season, therefore, the total mass of all seabed plants to have been washed up on Polish beaches may contain as much as 1.2 kg of mercury.

In the vicinity of seaside resorts (e.g., Sopot), macrophytobenthos is collected by relevant cleaning services and then taken away to be deposited in designated areas on the outskirts of the city. However, along stretches of coast less frequented by tourists (e.g., Oslonino and Rzucewo), it is left to accumulate rapidly in the coastal zone, where it serves as an additional source of food for crustaceans, molluscs and birds. What is more, mercury accumulated in beached phytobenthos can be re-emitted into the atmosphere, and this process is at its most intense in the summer season, when the highest temperatures and solar radiation levels are to be observed (Marks and Bełdowska 2001; Lindberg et al. 2005; Falkowska et al. 2013b).

## Conclusions

The urbanisation of the coastal zone has contributed to a rise in Hg concentration in macroalgae. Surface run-off entering bays together with the inflow of rainwater from sources such as storm drains, has led to higher mercury concentrations in *Cladophora* sp. in such areas in comparison with areas located further away. Among macroalgae, those which accumulated Hg the most effectively in the Southern Baltic were brown and red algae, a finding related to the chemical composition and construction of the cell wall of the thallus. The inflow of pollutants from river drainage areas also contributed to an increase in the concentrations of this metal in vascular plants, notably *Zostera marina*. Suspended particulate matter introduced to the sea via rivers, particularly in bays, was deposited close to the shore and became a source of Hg entering pore water and, in turn, vascular plants. Additionally, the capacity of vascular plants to accumulate chemical substances from pore water led to the accumulation of historical mercury deposits in the sediments. As a result, *Potamogeton pectinatus* demonstrated a bioconcentration factor 60 % higher than that of *Furcellaria lumbricalis*. Close to the outlets of the two rivers possessing the largest drainage areas in the Baltic (the Vistula and the Oder), no increased mercury concentrations were observed in macrophyta.

In the Polish coastal zone of the Southern Baltic, the reduction of Hg emissions into the environment has led to a decrease in concentrations of this metal in macrophyta. At the same time, however, the growing coverage of red algae on the seabed, stimulated by the increase in environmental quality and a prolonged vegetative season, results in both contemporary mercury from the land and historical deposits of the metal becoming rapidly introduced into the trophic chain. Seagrass beds provide an environment in which animal organisms thrive, often on consumption of phytobenthos (which accumulate Hg directly), and thus rapid growth of these plants may contribute to faster inclusion of Hg in the trophic chain. This is particularly important in areas where seagrass beds of *Zostera marina* are reviving, as their biomass was found to contain the most mercury. Therefore, although these macrophytae purify surrounding water and sediment, they are also responsible for the transfer of Hg, to more advanced organism on higher levels of the trophic chain.
